# Editorial Notes

**Published:** 1906-06

**Authors:** 


### Editorial Notes.

**University College Colston Society and the Bristol University.**

The seventh annual dinner of the University College Colston Society took place on March 31st under the presidency of Mr. J. W. Arrowsmith, and His Excellency the United States Ambassador (the Hon. Whitelaw Reid) and the Minister for Education (the Right Hon. Augustine Birrell) were the guests of the evening. The occasion is a memorable one, for Mr. Arrowsmith announced that those of them who had been working so hard for many years with the idea of establishing a university for Bristol had now determined to take the matter up in earnest. This is as it should be, for Bristol has waited long enough, and a general committee for this object is already in process of formation. Many influential persons have agreed to join this committee — the list containing the names of the Duke and Duchess of Beaufort, the Marquis of Lansdowne, the Marquis of Bath, Earl Ducie, Viscount St. Aldwyn, the Right Hon. Walter Long, Lord Fitzmaurice, Lord Winterstoke, Sir Frederick Wills and Sir John Dorington — and so we have good reason to hope that the success of the scheme is assured. But a university cannot be obtained without a considerable sum of money — we want about £200,000 — and it was a pleasing augury that Mr. Arrowsmith was able to announce that four gentlemen had generously offered contributions to the amount of £30,000— Lord Winterstoke and Mr. Joseph Storrs Fry £10,000 each, and Sir Frederick Wills and Mr. Francis James Fry £5,000 each. That is a good beginning; but we must continue to "think in thousands" if our hopes are to be realised. As Mr. Arrowsmith pointed out, it only requires twenty sums of £5,000 to raise the amount of money necessary. Some of this money will be required for the purpose of extending the present University College, and it is hoped that the site of the Blind Asylum may be acquired, as this would give the necessary accommodation.

Many people in England do not recognise what an enormous amount of money is spent on education by other states. We have been slow to appreciate the influence of education on commerce, and the State and people have been too willing to leave the matter to the care of the private individual. As the American Ambassador pointed out, the United States regard this matter as of national importance, and as an example he stated that New York State spent in 1905, out of the Treasury, State and local, something approaching £9,000,000 on education. Even with such huge sums as these at their disposal he thought that none of the American schools had money enough. In England, however, at present, as the Right Hon. Augustine Birrell said, we must rely upon the energy and generosity of our leading citizens, and he assured us that "no gifts were more embalmed or consecrated in the memory of a city than those which went to promote its highest education"—words which are worth remembering by those wealthy citizens to whom we must look for the large sum of money required.

We are much indebted to Professor Travers for his excellent pamphlet on the university problem, and would advise all those who are interested in the subject to carefully study its pages. A brief survey will at once convince the most sceptical that the position of Bristol is greatly inferior to that of other cities and towns in respect of its educational status. Thus in Liverpool we find in 1905 the value of the buildings and equipment was £310,000, the endowment (including scholarship grants) was £325,000, and the total income was £53,162. Against this we find Bristol in 1904 credited with £60,000 as the value of buildings and equipments, only £5,000 endowment, and £10,000 annual income. Another point which is brought out is the great lack of support in Bristol for its University College. Whereas Liverpool received in 1905 £12,800, Bristol received only £760 per annum on a mean for eight years. This want of contribution from local sources unfortunately has a secondary detrimental effect on the distribution of the Government grant, from which Liverpool receives £10,000 and Bristol £4,000.

The urgent need of Bristol is expressed in the fact that it is the only large city in England in which there is no university. Space will not allow us to mention in detail the many advantages which would accrue to Bristol by the establishment of a university, but some of the conclusions formed by Professor Travers may be stated. (1) The establishment of a university in Bristol will stimulate research, and will promote the development of higher education. (2) It will bring higher education more closely into touch with the commercial, industrial, professional and social life of Bristol and the West of England. (3) It will enable this city to provide university education for such persons as cannot afford to go elsewhere to obtain it, and will retain to the city many who will otherwise seek their education in some city in which a university is already established.

**Fifteenth International Medical Congress, Lisbon, 1906.**

Two members of the Society having been appointed delegates to represent our Society at this Congress, it becomes the duty of the Editor to give some report of the proceedings. The story of the English contingent is for the most part comprised in "the cruise of the *Ophir*," a twin-screw steamer of 6,814 tons and 10,000 horse-power, which left Tilbury Dock on April 12th, and after a journey of 2,688 miles, with a stay of six days on the Tagus opposite Lisbon, reached Tilbury again on the 29th. The list of passengers, comprising 250 names, was made up largely of medical men and members of their families, and the cruise was especially arranged for their convenience. In spite of adverse criticism, it has been a great success. It enabled those who wished to take a leading part in the arrangements of the Congress at Lisbon, and it also enabled them to see something of Tangier, Gibraltar, Vigo, and Oporto. The visit to Tangier is one which is not likely to be forgotten, and it is impossible to realise the scene without a visit. The present state of the land of the former conquerors of Southern Spain cannot be grasped without personal inspection.

The story of the Congress has been well told in the medical journals, and, with some minor inaccuracies, is substantially correct. The difficulties in ascertaining what was going on in the sections, and the difficulties of finding any given thing at the stated time were the same as at Madrid. It appears that the lapse of time and ideas of punctuality are scarcely understood throughout the Iberian peninsula. As regards the major functions of the Congress, everyone is agreed that everything was exceedingly well arranged, that it was well organised, successful, and enjoyable, and that much good work was done. The delightful entertainments gave universal satisfaction, and the fête at Villa Franca de Xira was one which has never been exceeded in enthusiasm and will never be forgotten by the recipients of so royal an ovation. The King and the stately Queen of Portugal did everything in their power to make the Congress a brilliant international success, and it must be a great satisfaction to the President, Conselheiro Costa Alemão, and his energetic, ubiquitous and genial Secretary-General, Professor Miguel Bombarda, to feel that their untiring exertions have been so well rewarded.

The English contingent was by no means an insignificant one, the names of Pavy, Dyce Duckworth, Barlow, Ferrier, Moore, Mayo Robson, and many others, who were mostly enrolled as Presidents of Honour, are sufficient to show that English medicine and surgery were well represented. One of the most noteworthy papers was that of Dr. F. W. Pavy, on his favourite subject, "The Pathogeny of Diabetes," in which he gave a terse account of his own life's work on this subject. Sir Dyce Duckworth gave a paper on a subject in which he has long taken an interest, "Chorea considered as Cerebral Rheumatism." The conclusions of this paper were as follows :—

1. That chorea (of Sydenham) is now regarded as a disease caused by an infective agency, and not as the result merely of shock or fright occurring in persons of neurotic instability.

2. That the peccant matter of rheumatism, which is now recognised as of microbic origin, is certainly the most frequent if not the sole cause of the infection.

3. That no other microbic element than this diplococcus has been discovered in those cases of chorea which are believed to be unconnected with rheumatism and independent of rheumatic influence.

4. That the infecting agent appears to be somewhat akin to but distinct from other varieties of streptococcus which induce ordinary forms of pyæmia.

5. That chorea is therefore to be regarded as a form of rheumatism involving the membranes and cortical structure of the brain, and is in fact cerebral rheumatism.

6. That there is a neurotic element in the pathogeny of chorea predisposing subjects thus impressed to this manifestation, amongst others, of rheumatism.

7. That chorea is thus a neuro-humoral disorder.

Dr. Logan, of Harrogate, read a paper on the Solution of some Neurological Problems, and Dr. Ferrier described his views on the Pathology of Tabes. Other papers were read by Dr. Mott, on Sleeping Sickness; Mr. Herbert J. Robson, of Leeds, on Appendicitis and on Pneumonia; Mr. D'Arcy Power, on Non-Malignant Ulcer; Dr. Edward Wolfenden Collins, on Myositis Ossificans; Dr. George F. Still, on Cerebral Palsy in Children; Dr. Bashford, on the Classification of the Sarcomata; Dr. Saundby, on the Treatment of Cirrhosis of the Liver; Mr. Reginald Harrison, on Surgical Interference in Medical Nephritis; Mr. Treacher Collins, on Intra-ocular Tuberculosis; and Professor Boyce, on Yellow Fever.

The cinematograph demonstration of various forms of Epilepsy, Athetosis, and other nerve defects, given by Dr. Chase, of Boston, was much appreciated, and we have since had an opportunity of again seeing those remarkable pictures reproduced in our own medical library.

Another interesting demonstration was that given by Dr. Sauerbach, who operates in a closed chamber in which the atmospheric pressure can be reduced to eleven millimetres of mercury. At this low pressure the chest of a patient may be opened without any risk of pulmonary collapse, the anæsthetic being administered with the head of the patient outside the chamber. Two or more assistants may work in the attenuated atmosphere of this chamber without discomfort, indeed the writer saw many operations on a dog performed in a very short time, the operator appearing to be in no discomfort and at no disadvantage from the confined space in which he was working.

The next congress is to be held at Budapest in the year 1909.

**Cerebro-Spinal Fever.**

A MEMORANDUM on this subject recently issued by Mr. W. H. Power is worthy of attention. Cerebro-spinal fever is not of rare occurrence in the United Kingdom. In the course of the last forty years this malady is known to have been prevalent in a considerable number of different localities in England and Wales, several of these local outbreaks having taken place in recent years. In some instances they have formed the subject of investigation by a Medical Inspector of the Local Government Board, whose report has usually been made public. Occurrences of cerebro-spinal fever have also been observed in Scotland and in Ireland. There is indeed, as will appear later, reason for belief that this disease is even less uncommon in this country than might be supposed from many newspaper references to single cases which have been reported from time to time. The term "spotted fever" appears to us to be altogether inappropriate, and should be left as formerly to be the synonym of typhus fever. Many cases of cerebro-spinal fever have no petechiæ or spots of any kind. It is now well known that an important aid to diagnosis may be found in examination of cerebro-spinal fluid, withdrawn from the lower part of the spinal canal by lumbar puncture, for the presence of the "diplococcus meningitidis intracellularis" of Weichselbaum, a micro-organism which is now generally regarded as the specific cause of cerebro-spinal fever.

Whether cerebro-spinal fever is spread by direct infection from person to person is matter of uncertainty; indeed, there is as yet no definite knowledge as to the way or ways in which its transmission may take place. Since, however, the possibility of direct personal infection cannot, on the evidence available, be excluded, it will be wise to endeavour to secure, as far as practicable, the isolation of the sick from the healthy. It will also be advisable to apply suitable measures of disinfection to premises that have been occupied by the sick, and to articles that may have been in relation with them. This subject received much attention at the Lisbon congress, when Dr. Judice Cabral (Lagos) gave quite a monograph upon the epidemic form, one of the most complete and thorough accounts of the disease founded on personal observation in a recent epidemic. Professor Anton Weichselbaum, of Vienna, gave evidence in favour of the view that the cavity of the naso-pharynx was the channel through which the micrococcus both found an entrance and was also conveyed from the sick to the healthy during the acts of sneezing, coughing, speaking, &c. The sporadic form was not uncommon, and cases might occur year after year, often without any apparent connection other than the fact of vicinity. Both speakers commented on the necessity for a bacteriological examination of the cerebro-spinal fluid to be obtained by lumbar puncture.

**British Electro-therapeutic Society.**

It is customary for some of the London Medical Societies to hold its meetings during the year in the provinces, and we are glad to find that the British Electro-therapeutic Society propose to hold their summer meeting this year at Clifton. The meeting will be held on Friday, July 27th, at the Clifton Down Hotel, under the presidency of Mr. J. Hall Edwards, the President of the Society, and it is hoped that any member of the profession interested in the subjects treated of will attend. Programmes of the proceedings and lists of papers to be read will be sent out as soon as the details are arranged. Papers which it is hoped will prove of interest to all members of the profession have already been promised by Dr. Lewis Jones, of St. Bartholomew's Hospital, and other prominent electricians. The Society was founded some few years ago by the medical officers in charge of the electrical departments of the various metropolitan hospitals, with the idea of bringing electrical methods of treatment and their usefulness more prominently before the profession, and at the same time to promote in every way the plan of keeping electrical treatment (including X-ray treatment) in the hands of qualified men. Dr. Reginald Morton and Dr. Kenneth Wills are the hon. secretaries of the Society.

**The Royal Sanitary Institute.**

THE arrangements for the annual meeting in Bristol, July 9th to 14th, are progressing satisfactorily, and the Health Exhibition in the Drill Hall will be open from the 9th to the 21st. A lecture on the "Relation of Heredity to Physical Degeneration" is to be given by Professor C. Lloyd Morgan, F.R.S., and the following set subjects are to be discussed in the various sections:—

*Section I.*—SANITARY SCIENCE AND PREVENTIVE MEDICINE.—"An International System of the Notification of the more serious Infectious Diseases," to be introduced by E. Walford, M.D., D.P.H.

*Section II.*—ENGINEERING AND ARCHITECTURE.—"The Construction of Isolated Homes for the aged poor *versus* the Workhouse," to be introduced by A. Saxon Snell, F.R.I.B.A.

*Section III.*—PHYSICS, CHEMISTRY, AND BIOLOGY.—"The Influence of Dust on Health," to be introduced by Philip Boobbyer, M.D.

*Conference of Municipal Representatives.*—"The Rational Extension of Modern Cities," to be introduced by Arthur Richardson, M.P.

*Conference of Engineers and Surveyors.*—"Rural Road Construction and Maintenance," to be introduced by R. Phillips, M.Inst.C.E.

*Conference of Medical Officers of Health.*—"Amendment of existing laws and the need of Additional Powers for greater efficiency in Public Health Administration."

*Conference of Veterinary Inspectors.*—"Milk Contamination in Collection and Transit," to be introduced by J. S. Lloyd, F.R.C.V.S.

*Conference of Sanitary Inspectors.*—"Advantages of Public Abattoirs," to be introduced by Geo. Anderson.

*Conference of Women on Hygiene.*—"Free Meals for 'Nursing Mothers,'" to be introduced by Miss Edith Evans.

*Conference on the Hygiene of School Life.*—"Instruction and Hygiene in Training Colleges," to be introduced by Miss Alice Ravenhill.

It will be seen that the subjects are all of much interest to the medical profession, and it is hoped that the medical element will be largely represented at the sectional as well as at the general meetings.

### Notes on Preparations for the Sick.

